# Magnitude of Birth Defects in Central and Northwest Ethiopia from 2010-2014: A Descriptive Retrospective Study

**DOI:** 10.1371/journal.pone.0161998

**Published:** 2016-10-05

**Authors:** Molla Taye, Mekbeb Afework, Wondwossen Fantaye, Ermias Diro, Alemayehu Worku

**Affiliations:** 1 Department of Anatomy, College of Health Sciences, Addis Ababa University, Addis Ababa, Ethiopia; 2 School of Dentistry, College of Health Sciences, Addis Ababa University, Addis Ababa, Ethiopia; 3 Internal medicine, School of Medicine, College of Medicine and Health Sciences, the University of Gondar, Gondar, Ethiopia; 4 School of Public Health, College of Health Sciences, Addis Ababa University, Addis Ababa, Ethiopia; Tabriz University of Medical Sciences, ISLAMIC REPUBLIC OF IRAN

## Abstract

**Background:**

Birth defects are defined as structural and functional defects that develop during the organogenesis period and present at birth or detected later in life. They are one of the leading causes of infant and child mortality, morbidity, and long term disability. The magnitude of birth defects varies from country to country and from race/ethnicity to race/ethnicity, and about 40–60% of their causes are unknown. The known causes of birth defects are genetic and environmental factors which may be prevented. For various reasons, there is lack of data and research on birth defects in Ethiopia.

**Objective:**

The major objective of this study is to estimate the magnitude of birth defects in Ethiopia.

**Subject and Methods:**

A hospital based, retrospective, cross sectional, descriptive study was conducted. The subjects were babies/children aged 0–17years who visited selected hospitals between 2010 and 2014. Fourteen hospitals (8 in Addis Ababa, 6 in Amhara Region) were selected purposively based on case load. A data retrieving form was developed to extract relevant information from record books.

**Results:**

In the hospitals mentioned, 319,776 various medical records of children aged 0–17years were found. Of these, 6,076 (1.9% with 95% CI: 1.85%–1.95%) children were diagnosed as having birth defects. The majority (58.5%) of the children were male and 41.5% female. A slightly more than half (51.1%) of the children were urban dwellers, while 48.9% were from rural areas. Among the participants of the study the proportion of birth defects ranged as follows: orofacial (34.2%), neural tube (30.8%), upper and lower limb (12.8%), cardiovascular system (10.3%), digestive system and abdominal wall (4.8%), unspecified congenital malformations (2.5%), Down syndrome (2%), genitourinary system (2%), head, face, and neck defects (0.4%), and others (0.3%). The trend of birth defects increased linearly over time [Extended Mantel-Haenszel chi square for linear trend = 356.7 (P<0.0001)]. About 275 (4.5%) of the cases had multiple (associated) birth defects and 5,801 (95.5%) isolated (single) birth defects. Out of the total birth defects, 6,018 (99%) were major and 58 (1%) minor.

**Conclusion:**

The magnitude of birth defects increased from 2010–2014. Orofacial and neural tube defects contributed about two thirds of the birth defects. There is an urgent need for registry and surveillance system strategies for intervention and control of birth defects in Ethiopia.

## Introduction

The development of the embryo is a complex process from the time of fertilization to the formation of all cells, tissues and organs. In early pregnancy, each body organ (system) has a critical period of organogenesis. Interference during this early pregnancy with intrinsic and extrinsic factor/s (i.e. parental and multifactoral effects) can lead to different forms of birth defects[1–3]. Birth defects are one of the leading causes of infant and child mortality, morbidity, and long term disability [4, 5]. Birth defects are defined as structural, functional, behavioral, and metabolic defects that develop during the organogenesis period and present at birth or detected later in life [2]. Birth defects (BDs) can be caused by genetic, chromosomal, environmental, and multifactoral effects, as well as micronutrient deficiencies or unknown etiological agents [6, 7].

It has been reported that etiologic agents (multifactoral effects, single genetic factors, and environmental factors) constitute 49.4%, 43.2%, and 7.4%, respectively, of the birth defects while the rest are unknown [7]. That is to say multifactoral effects play an important role in the causation of developmental abnormalities. It has been suggested that many BDs have genetic and environmental factors that contribute to the formation of a particular defect [8]. Other studies indicate that prenatal exposure to certain teratogenic agents has a high risk of having an infant with a BD [1, 9]. However, the risk of having a BD/congenital anomaly after exposure to an etiologic factor depends on the nature and amount of the agent, time, and duration of exposure, as well as the presence of concurrent exposures and genetic susceptibility of the embryo [10].

According to the literature, 94% of the BDs and 95% of deaths from BDs occur in low and middle income countries [11]. The major BDs occur in 2–3% live born infants [12–14] and 20% of still born fetuses [12]. Furthermore, 15–30% of infant and child hospital admissions are due to BDs[2]. In addition, infant mortality due to BDs has increased in both developed and developing countries [15, 16].

The prevalence of BDs varies widely from country to country, from region to region, and from race/ethnicity to race/ethnicity with a range of 1 to 4% [4, 17]. Neural tube and congenital heart defects, as well as orofacial and musculoskeletal defects are the most common birth defects occurring with high incidence rates [11].

Birth defect studies are important public health issues for planning and implementing prevention strategies and health services [17]. Planning for the BD program activities with clear aims, objectives and intended short, medium and long term outcomes are essential to reduce the burden of BDs. In addition, establishing a surveillance system as well as obtaining political support, financial aid and identifying geographical regions and understanding the health care system capacity are also important components to reduce the occurrence of BDs. Implementing preventing strategies based on BD data, linking children with BD to the health service and creating awareness about BDs and the uses of folic acid/multivitamins or nutritional status are necessary to reduce the events of BDs. It is also important to identify cases, establish data base and report to partners and responsible bodies. For many reasons, research on BDs and associated risk factors are not conducted adequately in Ethiopia. There has been no active surveillance and monitoring system at national, regional, or local levels, either. As a result, data and research capable of providing information on BDs have been scarce in Ethiopia, making a thorough investigation of the situation essential. So the purpose of this study was to describe and estimate the magnitude of BDs in Addis Ababa and the Amhara Region, by using the 2010–2014 hospital records as a starting point. The study also aimed at obtaining estimates of birth occurrence outcome and baseline information for further research.

## Subjects and Methods

### Study sites and setting

The study was conducted in 7 public and 7 private hospitals in Addis Ababa and the Amhara Region.

Addis Ababa, the capital city of Ethiopia, has 10 sub-cities with a population size of 3,273,001 (47.4% male, and 52.6% female) [18]. The fertility rate of Addis between 2007 and 2012 was estimated at 2.1% per woman, and over 50% of the people live below the poverty line. As the melting pot of all cultures and ethnic groups of the country, the city is subject to population increase through constant migrations. All of the hospitals in the city, included in this study except Cure International Children’s Hospital provide delivery services in addition to other health services [19].

With an estimated population of 20,399,004 (50.1% male, 49.9% female), Amhara is the second largest region in the country. The majority of the people lives below the poverty line and still has an annual growth rate of 2–3%. Most of the Amhara people are Christians with a good number of Muslims, and some followers of other faiths. All of the hospitals in the regional state provide delivery as well as other health services.

### Selection of study hospitals

A total of fourteen hospitals, eight in Addis Ababa (4 public, 4 private), and six in the Amhara Region (3 public, 3 private) were purposely included in the study on the basis of case load. Because our objectives were to show that the necessity of routinely collected medical records/data which are essential for research purposes as well as its validation for the establishment of a better strategic plan in the health service management, registry, evaluation, surveillance and controlling systems. In addition, this study is capable to provide information about birth defects situation in the country. The hospitals had various specialized departments or units which provide various services for neonates, children, and adults. In all the study hospitals there are Pediatricians, Surgeons, Obstetricians/Gynecologists, Internists, Midwifes, Nurses and other health professionals who are working permanently. In addition, some hospitals have Neonatologists, Plastic Surgeons, and Consultants. Relevant data were extracted from the selected public and private hospitals.

### Design of the study

The design of the study was a retrospective medical record review.

### Data collection

Data were collected from hospital records from October 2014-July 2015. All case records (of 0–17year old children’s) were reviewed carefully. Cases older than 17 years and cases from the Amhara Region referred to Addis Ababa were excluded from the study in order to avoid repetition. All birth defect (i.e. both external and internal body structural defects) information seen in the patients’ charts/medical history record books were collected by using a verbatim description and a semi-structured checklist. The checklist contained study participants’ hospital chart number/medical record book serial numbers, region, residence, age, sex, ethnicity, diagnosis/type of birth defect, year of diagnosis (i.e. used to describe/write down the available information in the case chart/medical history record book), and the presence of maternal history of risk factors, such as history of alcohol intake, diabetes, cigarette smoking, family history of birth defects, drug, and folic acid intake. The data collectors gathered the preceding information by writing out the factors. The data were collected at delivery wards, neonatal units, children’s clinics, and at selected cleft lip and palate centers by the primary investigator, midwives, and nurses working at the study hospitals. During data collection period Pediatricians and Neonatologists were consulted when there is an unclear diagnosis. Moreover, diagnoses were excluded when it was unclear and not confirmed by pediatricians/experienced specialists. The proportion of children with BDs was calculated by dividing the number of birth defect cases (numerator) by the total number of children visiting the hospitals or treated for any problem (denominator) in the same period. Study cases with more than one birth defect were counted once. Cases were included if they met the case definition of birth defects. Birth defects whether they are major or minor are defined as anatomical structural and functional defects present at birth and are of prenatal origin. Major birth defects are defined as structural and functional congenital anomaly that have health and social impacts upon the affected person and need medical interventions, while minor birth defects are anatomical structural defects that have minimal social and health effects and require no medical interventions [20].

### Data handling and analysis

Although BD’s were recorded in the hospitals before 2010, we decided to start from this year in order to limit our investigation to five years. Data entry, cleaning, error checking, and analysis were conducted by using SPSS, version 21. Descriptive analysis (frequency) was calculated for the variables. Proportions were calculated with a 95% confidence interval (CI). Mantel-Haenszel summary Odds Ratio and Extended Mantel-Haenszel chi square was carried out to determine the linear trend of birth defects over time. The outcome of interest was birth defects, and the independent variables were sociodemographic characters, such as children’s age, sex, residence/region, presence of risk factors, and year diagnosed (2010–2014).

### Ethical clearance

Before starting the study, ethical approval and waiver was obtained from the National Research Ethics Review Committee, Ref. No. 3.10/781/07, dated October 16, 2014; Addis Ababa University, College of Health Sciences Institutional Review Board, Meeting Ref. No. 060/14, dated June 12, 2014; HARI-ALERT Ethical Review Committee, Project Reg. No. PO58/14, dated December 23, 2014; The Addis Ababa City Administration Health Bureau Ethical Clearance Committee, Ref. No. A/A/H/B/1972/25, dated 27/10/2014; Amhara National Regional State Health Bureau Regional Health Research Laboratory Center, Ref. No. ላብ/20/4963, dated 27/01/2007(Ethiopian Calendar). Supportive letters were written to all zonal health departments and study hospitals by health bureaus. The ethical and supportive letters were submitted to all study hospital administrators. Data collection began after permission was obtained from hospital administrators/managers. Information gathered from record books were kept in a secured and locked cabinet in order to maintain confidentiality. The study participants were not present during data collection. Since the study obtained ethical clearance letter and waiver from Ethics Review Boards, the study hospitals agreed and stamped seal at the bottom of the ethical clearance letter.

## Results

From 2010–2014, there were 319,776 various medical records of children aged 0–17years. Out of these, 6,076 (58.5% male, 41.5% female), (1.9% with 95% CI: 1.85%–1.95%) had BD’s. Most of the children were below 1year of age (Table 1). Male children who had BDs significantly outnumbered the females. Three thousand one hundred and two (51.1%) of the participants lived in urban areas, while 2974 (48.9%) in rural settlements. The socio-demographic characteristics of the cases are shown in Table 1.

**Table 1:** Socio-demographic characteristics of study subjects in Addis Ababa and Amhara Region, 2016.

Variable	Addis Ababa		Amhara Region		Total	
	Number	(%)	Number	(%)	Number	(%)
**Gender**						
Male	2765	58.9	790	57.2	3555	58.5
Female	1929	41.1	592	42.8	2521	41.5
**Age**						
0–1year	2855	60.8	613	44.4	3468	57.1
2–6year	1173	25	375	27.1	1548	25.5
7–11year	418	8.9	173	12.5	591	9.7
12–17year	248	5.3	221	16	469	7.7
**Residence**						
Urban	2701	57.5	401	29	3102	51.1
Rural	1993	42.5	981	71	2974	48.9
**Region originated**						
Addis Ababa	2133	45.4	-	-	2133	35.1
Oromya	1809	38.5	4	0.3	1813	29.8
Amhara	311	6.6	1332	96.4	1643	27.0
Somali	71	1.5	-	-	71	1.2
Afar	9	0.2	44	3.2	53	0.9
Harar	52	1.1	-	-	52	0.9
Tigray	44	0.9	1	0.1	45	0.7
Dire-Dawa	23	0.5	-	-	23	0.4
Benishangul-Gumuz	7	0.1	1	0.07	8	0.1
Gambella	8	0.2	-	-	8	0.1
SNNP	227	4.8	-	-	227	3.74
**Ethnicity**						
Amhara	242	5.2	130	9.4	372	6.1
Oromo	251	5.3	-	-	251	4.1
Somalie	41	0.9	-	-	41	0.7
Tigrie	13	0.3	-	-	13	0.2
Guragie	16	0.3	-	-	16	0.3
Hadya	3	0.1	-	-	3	0.0
Others	56	1.2	2	0.14	58	0.95
Unknown/Unrecorded	4072	86.7	1250	90.4	5322	87.6

SNNP = Southern Nationalities Nations and Peoples

As indicated in Table 2, of the total birth defects, the most frequent was orofacial (34.2%), followed by neural tube (30.8%), upper and lower limb (12.8%), cardiovascular system (10.3%), digestive system and abdominal wall (4.8%), unspecified congenital malformations (2.5%), Down syndrome (2%), genitourinary system (2%), head, face, and neck defects (0.4%), and others (0.3%).

**Table 2:** Frequency distribution of birth defects by sex 2010–2014 in Addis Ababa and Amhara region, 2016.

Variables	Male(n = 3555)		Female(n = 2521)		Total(n = 6076)	
	Number	%	Number	%	Number	%
Orofacial Clefts	1278	36	798	31.7	2076	34.2
Neural Tube Defects	1010	28.4	863	34.2	1873	30.8
Upper and Lower Limb Defects	525	14.8	247	9.8	772	12.7
Cardiovascular system defects	309	8.7	317	12.6	626	10.3
Digestive system and abdominal wall defect	166	4.7	123	4.9	289	4.8
Down Syndrome	57	1.6	67	2.7	124	2.0
Genitourinary system defects	108	3.0	15	0.6	123	2.0
Unspecified Congenital Malformation	82	2.3	68	2.7	150	2.5
Head, Face and Neck defects	10	0.3	14	0.6	24	0.4
Others*	10	0.3	9	0.4	19	0.3

* Amniotic band, Chest deformity, Congenital diaphragm hernia, Congenital Ptosis; Congenital micro-ophthalmia, Conjoined twin with congenital malformation, fistula communicating between sinus tracts, Hemangioma, Osteogenesis imperfecta, Congenital perineal fistula & Pulmonary atresia with intact ventricular septum.

Among the orofacial clefts (OFCs), the proportion of cleft lip, cleft lip and palate, and cleft palate was 70.2, 21.2, and 8.3%, respectively (see Table 3). The frequency of orofacial clefts by laterality showed that unilateral cleft lip was the most frequent, (63.2%), and bilateral cleft palate the least frequent, (0.7%), (see Table 4). As far as neural tube defects (NTDs) are concerned, spinal bifida, including lumbar, sacral, thoracic, and cervical spinal bifida, with or without mengocele/menigomyelocele, was more frequent, (44.6%) than anencephaly, (8.7%) while, encephalocele was the least frequent, (1.3%), (see Table 5).

**Table 3:** Frequency distribution of orofacial clefts by type from 2010–2014 in Addis Ababa and Amhara region, 2016.

Variable	Frequency(n = 2076)	Percent
Cleft lip	1463	70.5
Cleft lip and palate	440	21.2
Cleft palate	173	8.3

**Table 4:** Revealing frequency of orofacial clefts by laterality, in Addis Ababa and Amhara region from 2010–2014.

Type of cleft by laterality	Frequency(n = 2076)	Percent
Unilateral cleft lip	1311	63.2
Bilateral cleft lip	116	5.6
Unilateral cleft lip and palate	296	14.3
Bilateral cleft lip and palate	99	4.8
Unilateral cleft palate	108	5.2
Bilateral cleft palate	15	0.7
Others*	131	6.2

* Uncategorized cleft lip; uncategorized cleft lip and palate; uncategorized cleft palate, midline cleft lip; midline cleft lip and palate, and cleft lip, cleft palate with other birth defects.

**Table 5:** Frequency of Neural Tube Defects from 2010–2014 in Addis Ababa and Amhara region, 2016.

Variable	Frequency(n = 1873)	Percent
Spinal bifida(lumbar, sacral, thoracic and cervical)	836	44.6
Hydrocephaly	653	34.9
Anencephaly	163	8.7
Spinal bifida with hydrocephaly	139	7.4
Encephalocele	25	1.3
Anencephaly with spinal bifid	20	1.1
Others*	37	2.0

* Spinal bifida with other body system birth defects; Anencephaly with other system birth defects; hydrocephaly with other system birth defects and Encephalocele with other system birth defects.

The linearly increasing trend of the proportion of BDs over the five year period was 1.14, 1.65, 1.69, 1.75, 2.83%, respectively [Extended Mantel-Haenszel chi square for linear trend = 356.7 (P<0.0001)], (see Table 6). About 275 (4.5%) of the cases had multiple (associated) BDs, and 5,801 (95.5%) had isolated (single) BDs. On the other hand, out of all types of BDs, 6,018 (99%), were major BDs, and 58 (1%) minor BDs. The study also showed that the distribution of BDs varied among the study hospitals, (see Table 7).

**Table 6:** Linear trend of birth defects from 2010–2014 in Addis Ababa and Amhara region, 2016.

Year	No of children	No of children with birth defect	Proportion	Mantel-Haenszel Summary Odds Ratio
2010	31234	355	1.14	1
2011	68041	1121	1.65	1.46
2012	73820	1251	1.69	1.50
2013	74232	1301	1.75	1.55
2014	72449	2048	2.83	2.53

Extended Mantel-Haenszel chi square for linear trend over time = 356.7 (P<0.000l).

**Table 7:** The percentage distribution of BDs 2010–2014 among the study hospitals in Addis Ababa and Amhara Region, 2016.

Hospitals name and ownership by study sites	Number	Percent
**Addis Ababa**		
Addis Hiwet General Hospital℗	740	12.2
ALERT Hospital*	159	2.6
Cure International Children’s Hospital℗	787	13
MCM Korean Hospital℗	357	5.8
Betsegah Special Women’s and Children’s Hospital℗	50	0.8
Tikur Anbesa General Specialized Hospital*	1264	20.8
Yekatit 12 Hospital*	224	3.7
Zewditu Memorial Hospital*	1113	18.3
**Amhara Region**		
Desse Referral Hospital*	308	5.1
Felegehiwet Referral Hospital*	192	3.2
Gamby Teaching Medical Sciences College Hospital℗	371	6.1
University of Gondar Teaching Hospital*	181	3
Ibex General Hospital℗	138	2.2
Selam General Hospital℗	192	3.2

* Public Hospitals

^**℗**^ Private Hospitals

The proportion of the BDs observed in the two data sites, Addis Ababa and the Amhara Region indicated that they bore 77.3% and 22.7% of the burden, respectively. This difference may be due to the fact that cases across the country were referred to Addis Ababa.

## Discussion

The findings of this study showed an increasing trend of birth defects (BDs) over time. It may be the first BDs study conducted on Ethiopian children. Our aim was to describe the situation of BDs in Ethiopia, particularly in Addis Ababa, the capital city of Ethiopia, and the Amhara Region. In Ethiopia, BDs has not been given attention despite the fact that understanding the prevalence and having knowledge on BDs etiology is an important public health issue, which may give insight to concerned bodies so they develop strategic plans for identifying possible causes, screening early pregnancies, and implementing intervention activities.

The over all proportion of BDs in this study was 1.9%, i.e. 19 per 1000 children, which is close to the finding (20.3/1000 births) of a study conducted in Uganda by Ndibazza, et al., [21]. However, various other studies showed that the prevalence rates of BDs ranged from 1.5% to 3% [2, 17, 22, 23]. Some studies also indicated prevalence rates of BDs ranging between 3% and 5% [24–26]. The frequency and range of BDs prevalence differ across regions/countries of the world due to differences in the methodology used by reasearchers, for instance, population based and hospital based studies [20, 27]. In Africa, there is lack of BDs data because of poor documentation, limited diagnosing capacity of health service providers, and lack of resources [21]. Except few data on orofacial clefts, there has been no research-based information in Ethiopia at local and national levels. Therefore, this high figure indicates the necessity of an immediate action that Ethiopia has to take. In addition, this figure should also be considered as a minimal estimate of BDs because the study was done against challenges, such as ascertainment, recording, and classification because there is no birth defect registry system in Ethiopia.

Our result is comparable with the findings of a study conducted in Egypt by Shawky and Sadik [2] but lower in frequency than that of a study conducted in Tanzania by Mashuda et al., [28]. This lower rate could be due to sample size and study design differences, that is, in Mashuda et al., [28] the participants were 445 infants, while ours were 6076 cases. Moreover, a study in China showed a higher prevalence rate (156.1/1000 births) [4] as compared to the present study. Similarly, a study in Metropolitan Atlanta reported such high rates as 323, 266, and 266 for each 10,000 live births among non-Hispanic whites, non-Hispanic blacks, and the Hispanic people, respectively [29]. Furthermore, a higher prevalence rate of birth defects was reported in Australia by Riley [26].

In our study, the highest proportion of BDs was OFCs (34.2%) followed by NTDs (30.8%) while the least frequent BDs observed was aminotic band and tongue tie (0.03% and 0.02%), respectively. Among the OFCs, unilateral cleft lip was the most frequently observed defect in this study. These results are in agreement with the findings carried out in Ecuador by Gonzalez-Andrade and Lopez-Pulles [30]. In contrast to our findings, other studies conducted in Romania found higher prevalence of congenital heart defects (33.06%), followed by respiratory tract defects [31]. A recent study in Arctic Russia found a higher prevalence (8.7%), of congenital malformations and deformations of the musculoskeletal system, followed by (4.3%) congenital malformations of the urinary system [6]. Muga et al., [32] in Kenya, found 33.9% prevalence rate of the musculoskeletal system defects, followed by 28.1% of the central nervous system defects which is different from our findings. The differences in prevalence may be due to genetic factors or the existence of multifactoral effects in the countries the studies were carried out. In the NTDs, we found 44.6% of spinal bifids, followed by hydrocephaly (34.9%), and anencephaly (8.7%). In contrast to our’s, a study conducted in China by Zhang et al., [4], found out a 10.6 per 10,000 births prevalence rate for spinal bifida, and 2.7 per 10,000 births for encephalocele. This difference may be due to a better recording system in China than in Ethiopia and differences in the methodologies employed.

In the present study, the proportion of BDs by study sites varied by being 75.5% for Addis Ababa and 22.5% for the Amhara Region. This difference may be due to the fact that more cases were referred to Addis Ababa. The proportion of BDs was slightly higher (51.1%) in urban than (48.9%) in rural areas. This variation in proportion may be due to higher exposure to risk factors in urban than in rural areas or may be due to life-style differences between urban and rural areas. The proportion of BDs for urban and rural Romania was reported as 63.4% and 36.4%, respectively [31]. On the contrary, a study carried out by Fan et al., [15] in China from 2000 to 2010 showed that prevalence rate increased from 1.0% to 1.05% in rural areas and from 0.68% to 0.91% in urban areas. Another study in China revealed that the rate of BDs was 179.4 per 10,000 births in rural areas and 124.6 per 10,000 in urban areas [4].

According to our study, more male children (58.5%) were observed with BDs than female ones (41.5%). Similarly, Mashuda et al., from Tanzania reported that 54.6% of the male and 44.9% of the female children were observed with congenital anomalies [28]. Furthermore, Bakare et al., in Nigeria and Zhang et al., in China pointed out that the proportions of BDs were 51.3% and 48.7%, and 54,9% and 38.7% for male and female, in that order [33], [4].

In our study, it was observed that BDs were significantly increasing in Ethiopia from 2010 to 2014. The proportion of BDs for the years 2010–2014 was 1.14%, 1.65%, 1.69%, 1.75%, and 2.83%, respectively. That is, there was a significantly increasing linear trend over time [Extended Mantel-Haenszel chi square for linear trend = 356.7 (P<0.0001)]. This increase in proportion may reflect the improvement of recording of hospitals, diagnosing capacity improvement, and also real increasing rates that need public health action. According to the literature, BDs prevalence from 1973 to 2011 increased from 23.5/1000 to 46.3/1000 live births plus stilbirths in Arctic Russia [6]. This difference suggest that the present study subjects lack folate and are malnourished due to famine. Other similar studies conducted in China from 2000 to 2010 [15] and in Turkey from 2000 to 2004 [24] showed increasing trends of congenital anomalies prevalence rates.

Over all, the most significant findings of this study are that isolated BDs constitute 95.5% and multiple ones 4.5%. This, however, is unlike that of a study conducted in Egypt by Ahmed et al., [34] which found out that 69% of the anomalies were isolated and 31% multiple congenital. Another study conducted in Nigeria showed that 87.8% were single system congenital anomalies and 12.2% multiple system congenital anomalies [27]. In the study in Nigeria, central nerveous system anomalies were the most dominat congenital anomalies [27].

We found that 99% of the birth defects were major and only 1% minor anomalies. However, a study conducted in Brazil showed that 66% of the malformations were minor [23]. In addition, a study carried out in Kenya revealed that 1.5% of the anomalies were major birth defects [32]. These widespread differences may be due to misdiagnosis and ascertainment problems or genetic variations.

Since ours is a descriptive, retrospective study conducted by using hospital based medical records of cases, we didn’t focus on the etiology of BDs and associations between risk factors and outcomes because of limited information in the record book from which we collected the data. According to the literature, however, the causes for about 40–60% of BDs are unknown [28, 34–36].

In conclussion, these findings suggest that there is an increasing burden of BDs in Ethiopia. The findings reflect that sustainable surveillance and registry systems are mandatory for intervention activities. In this respect, the current study might fill an important information gap on BDs in Ethiopia.

## Abbreviations

BDs: birth defects
OFCs: orofacial clefts
NTDs: neural defects
SNNP: southern nations nationalities and people
CI: confidence interval
